# Electrochemically Exfoliated Graphene-Like Nanosheets for Use in Ceramic Nanocomposites

**DOI:** 10.3390/ma13112656

**Published:** 2020-06-11

**Authors:** Rosalía Poyato, Reyes Verdugo, Carmen Muñoz-Ferreiro, Ángela Gallardo-López

**Affiliations:** 1 Instituto de Ciencia de Materiales de Sevilla, ICMS (CSIC-US), Américo Vespucio 49, 41092 Sevilla, Spain; 2 Departamento de Física de la Materia Condensada, ICMS, CSIC-Universidad de Sevilla, Apdo. 1065, 41080 Sevilla, Spain; ireyesvmz@gmail.com (R.V.); cmunoz7@us.es (C.M.-F.); angela@us.es (Á.G.-L.)

**Keywords:** graphene, electrochemical exfoliation method, 3YTZP, ceramic nanocomposites, planetary ball milling, SPS, Raman spectroscopy, electron microscopy, Vickers indentations

## Abstract

In this work, the synthesis of graphene-like nanosheets (GNS) by an electrochemical exfoliation method, their microstructural characterization and their performance as fillers in a ceramic matrix composite have been assessed. To fabricate the composites, 3 mol % yttria tetragonal zirconia (3YTZP) powders with 1 vol % GNS were processed by planetary ball milling in tert-butanol to enhance the GNS distribution throughout the matrix, and densified by spark plasma sintering (SPS). According to a thorough Raman analysis and SEM observations, the electrochemically exfoliated GNS possessed less than 10 graphene layers and a lateral size lower than 1 μm. However, they contained amorphous carbon and vacancy-like defects. In contrast the GNS in the sintered composite exhibited enhanced quality with a lower number of defects, and they were wavy, semi-transparent and with very low thickness. The obtained nanocomposite was fully dense with a homogeneous distribution of GNS into the matrix. The Vickers hardness of the nanocomposite showed similar values to those of a monolithic 3YTZP ceramic sintered in the same conditions, and to the reported ones for a 3YTZP composite with the same content of commercial graphene nanosheets.

## 1. Introduction

Since the first isolation of single-layer graphene in 2004 by the mechanical exfoliation of graphite —the “Scotch tape” method [1]—its unique properties have motivated a continuous growth in research activity. It has been considered as a feasible candidate for applications in fuel cells, composites, electronic devices, sensors, and photodetectors [2].

In the last decade, graphene has mainly been synthesized using two different approaches: bottom-up, in which graphene is grown from small molecular carbon precursors, and top-down, in which graphene is exfoliated from graphite as parent material [2]. Among the bottom-up approaches, the chemical vapor deposition (CVD) technique is the most popular way of deposition of graphene films on metal foils or silicon substrates [3,4,5]. Together with epitaxial growth [6], these are methods that allow the formation of high-quality, large area graphene, encouraging its application in highly flexible and conducting films. However, these methods present drawbacks such as high manufacturing costs or the requirement of sophisticated equipment, high temperatures and expensive substrates [3]. On the other hand, when top-down approaches such as mechanochemical synthesis [7,8] or liquid phase exfoliation [9,10] are simple, cost-effective and easily scalable, they have been presented by different authors such as suitable methods for graphene mass production [7,11]. The synthesis of graphene oxide by the mechanochemical method has also been reported [12]. The main disadvantage of these techniques is that the obtained structures can have a greater number of defects than the ones that originate from bottom-up methods.

Electrochemical exfoliation has had a strong impact on the development of techniques to obtain graphene because it provides an economical, simple and fast way to produce it. It is easily reproducible because it can be performed under environmental conditions, and toxicity-free components that can be easily removed after the process are used. In addition, the use of graphite as starting material, together with the good results obtained from this process, reduce the cost of producing graphene, resulting in an efficient and affordable method for the scientific community [13]. Moreover, some studies have reported the production of high-quality thin graphene sheets with lateral sizes up to 30 μm by electrochemical exfoliation of graphite [10,11,14]. However, when this type of synthesis technique is used, it is not easy to generate single-layer graphene, and graphene nanosheets (GNS) are usually obtained. Thus, after the synthesis step, it is essential to characterize the nanostructures in order to assess the lateral dimension, the number of graphene layers and the possible presence of defects created during the synthesis process [7,8,10]. In recent years, new approaches for the electrochemical exfoliation technique have been suggested in order to improve the yield [11,14] and to promote the obtaining of mostly single- and few-layer graphene sheets [15].

Among the different applications of graphene, its use as a filler in composite materials has awakened the interest of the scientific community in the last years, owing to the relevant properties that these nanostructures impart to most materials [2,16]. In the case of ceramics, a strong interest has been generated in the development of advanced ceramics in which the presence of graphene as a second phase improves their fracture toughness and electrical conductivity [17,18]. However, these composite materials present a processing challenge due to graphene’s strong tendency to agglomerate, as a consequence of its high surface area. This negatively affects the properties of the composite, so advanced processing techniques are usually needed [19,20]. Among the advanced ceramics, 3 mol % yttria tetragonal zirconia (3YTZP) presents a remarkable technological interest because of its excellent mechanical properties, such as Young’s modulus, fracture toughness and hardness, as well as its chemical stability [21]. Recent studies about 3YTZP composites with graphene have reported enhancements on properties as fracture toughness or flexure strength for very low additions of graphene nanostructures [22,23].

Most of the published studies about graphene-ceramic composites generally use commercially acquired graphene nanosheets. Although promising results in terms of enhancement of mechanical and electrical properties have been reported for composites with cost-effective graphene nanoplatelets prepared using advanced powder processing techniques [20,23], the best results have been obtained in composites with thinner and more expensive graphene nanosheets or few-layer graphene [24,25,26]. This could hinder the industrial application of these composite materials due to the high manufacturing costs. In this context, the search for cost-effective synthesis techniques to obtain graphene nanosheets for its application in ceramic nanocomposites is very necessary.

In this work, the synthesis of graphene-like nanosheets has been assessed by means of a simple, cost-effective and fast electrochemical exfoliation technique, using graphite as parent material. After a detailed characterization of the as-synthesized nanosheets by Raman spectroscopy and electron microscopy observations, they were incorporated as filler in a 3YTZP matrix nanocomposite. Powders with 1 vol % GNS were processed by planetary ball milling in tert-butanol to enhance the GNS distribution throughout the matrix, and densified by spark plasma sintering (SPS). The quality and level of defects of the GNS in the composite were assessed by Raman spectroscopy. The microstructure and hardness of the obtained nanocomposite was analyzed and compared to the reported ones for 3YTZP composites prepared with commercial nanosheets.

## 2. Materials and Methods

### 2.1. Graphene Synthesis and Characterization

The graphene-like nanosheets were obtained by the electrochemical exfoliation method [10], using a graphite bar (1 cm diameter, 10 cm long, Goodfellow Cambridge Ltd., Huntingdon, UK) and a platinum wire acting as anode and cathode, respectively. The ionic solution was prepared by taking 1.3 mL of sulphuric acid (95–98%, Panreac, Castellar del Vallès, Spain) and diluting in 100 mL of DI water. The platinum wire and the graphite bar were immersed into the ionic solution with a separation of 5 cm, and the electrochemical exfoliation process was carried out by applying DC bias from 1 to 10 V, with steps of 1 V every ten minutes during a total time of 1.5 h. After this time, 10 V were applied for 30 min. Continuous magnetic agitation was applied during the whole exfoliation process.

After the exfoliation process, the suspensions were washed with DI water and isopropyl alcohol by vacuum filtration using 200 nm pore filter alumina membranes (Whatman, Maidstone, UK) and centrifuged (model SIGMA 3-30KS, Sigma Laboratory Centrifuges, Osterode am Harz, Germany) at 8500 r.p.m. for 15 min to remove graphite aggregates. The suspensions were frozen with liquid nitrogen and freeze-dried for 48 h at −80 °C in order to avoid re-agglomeration of the obtained nanosheets during drying (Cryodos-80, Telstar, Terrasa, Spain).

Raman spectroscopy and high-resolution scanning electron microscopy (HRSEM, S5200, Hitachi High-Technologies Corp., Tokyo, Japan) were used to characterize the number of layers, morphology and size distribution of the as-synthesized GNS. To that end, a few droplets of GNS suspension in isopropyl alcohol were deposited on a glass slide for Raman spectroscopy or on a Cu transmission grid with C coating for HRSEM inspection after drying. At least 10 Raman spectra were acquired on the electrochemically exfoliated GNS using a dispersive microscope Raman Horiba Jobin Yvon LabRam HR800 (ICMS), with a green laser He-Ne (532.1 nm) at 20 mW. The first-order (from 1000 to 2000 cm^−1^) Raman spectra were fitted to a sum of five functions: two Gaussian and three pseudo-Voigt functions. In the second-order spectra (from 2250 to 3300 cm^−1^) three Lorentz and three pseudo-Voigt functions were used. The fits were carried out using the OriginLab software (OriginPro 2019, OriginLab Corporation, Northampton, MA, USA).

### 2.2. Nanocomposite Processing and Characterization

Composite powders with 1 vol % GNS were prepared using the electrochemically exfoliated nanosheets and commercial 3YTZP powders (40 nm particle size, TZ-3YB-E, Tosoh Europe B.V, Amsterdam, The Netherlands), which were previously annealed at 850 °C for 30 min in air. Planetary ball milling (Pulverisette 7 classic line, Fritsch, Idar-Oberstein, Germany) was used to homogenize the powders in a 10 w/w% tert-butanol (t-BuOH)/water mixture at 700 r.p.m. for 15 min. A 45 mL zirconia jar and seven 15 mm diameter zirconia balls were used. After drying on a rotary evaporator, the composite powders were homogenized in an agatha mortar and spark plasma sintered at 1250 °C for 5 min, with an applied pressure of 75 MPa and heating and cooling ramps of 300 and 50 °C/min, respectively (SPS model 515 S, Dr. Sinter, Inc., Kanagawa, Japan). A sheet of graphite paper was placed between the powders and the die/punches to both ensure their electrical, mechanical and thermal contact and also for an easy removal. The temperature was continually monitored by means of an optical pyrometer focused on the side of the graphite die. Cylindrical samples with 10 mm diameter and 2 mm thickness were obtained. The surface graphite paper from the SPS molding system was manually eliminated by grinding.

To account for possible structural modifications of the graphene-like nanosheets after the composite powder processing and sintering, at least ten Raman spectra were acquired on the obtained powders after planetary ball milling, and on the fracture surface of the sintered composite. The first- and second-order Raman spectra were fitted to the functions described in Section 2.1. The density of the composite was determined with the Archimedes’ method using distilled water as the immersion medium. The theoretical density was calculated by the rule of mixtures taking the density of the 3YTZP and the GNS as 6.05 g/cm^3^ and 2.2 g/cm^3^, respectively. Scanning electron microscopy (SEM) using backscattered electrons (BSE) for imaging (FEI-Teneo, FEI, Thermo Fisher, Cambridge, MA, USA) was used to analyze the dispersion of the GNS in the ceramic matrix. This microscope has two in-lens detectors which allow obtaining high resolution images at short work distances. Polished in-plane (i.p.) and cross-section (c.s.) surfaces were analyzed to account for the existence of any structural anisotropy on the composite. The grain size of the ceramic matrix was estimated from SEM images acquired on polished c.s. surfaces previously annealed in air for 15 min at 1150 °C. The planar equivalent diameter, d = 2(area/π)^1/2^, namely the diameter corresponding to a circle with the same area as the measured grain, was taken as a measure of the grain size, averaging 200 to 300 grains, according to UNE-EN ISO 13383-1:2016 standard. The software packages ImageJ and OriginLab were used to determine the relevant parameters. The fracture surface of the composite was also examined by HRSEM (HRSEM, S5200, Hitachi High-Technologies Corp., Tokyo, Japan).

The hardness of the nanocomposite was estimated from standard Vickers micro-indentations (Vickers Duramin indenter, Struers, Copenhagen, Denmark) performed on the mirror polished i.p. and c.s. surfaces. These two orientations were evaluated to account for any possible anisotropy effects. Ten indentations were performed on each surface with 1.96 N applied load during 10 s. The hardness values were calculated following the equation: H_V_ (GPa) = 1854.4 P/D^2^, where P is the applied load in N and D the average diagonal of the imprint in μm.

## 3. Results and Discussion

### 3.1. Microstructural Characterization of the Graphene Nanosheets

**Figure 1:**
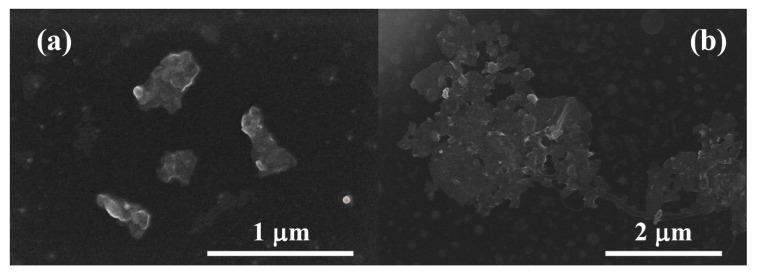
High-resolution scanning electron microscopy (HRSEM) images for the electrochemically exfoliated graphene nanosheets, drop-casted on a Cu transmission grid (**a**) Isolated nanosheets; (**b**) agglomerated nanosheets.

Typical HRSEM micrographs (acquired in Secondary Electron Image mode) for the electrochemically exfoliated nanosheets are shown in Figure 1. It can be observed that some nanosheets present a lateral size lower that 1 μm (Figure 1a). However, they show a strong tendency to agglomerate (Figure 1b), resulting in GNS interconnections with a lateral size of several microns.

**Figure 2:**
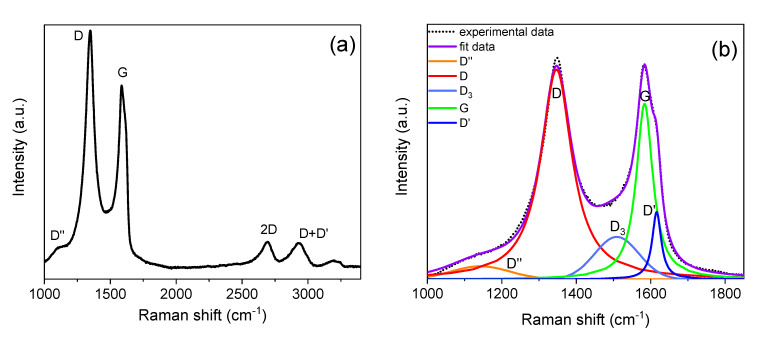

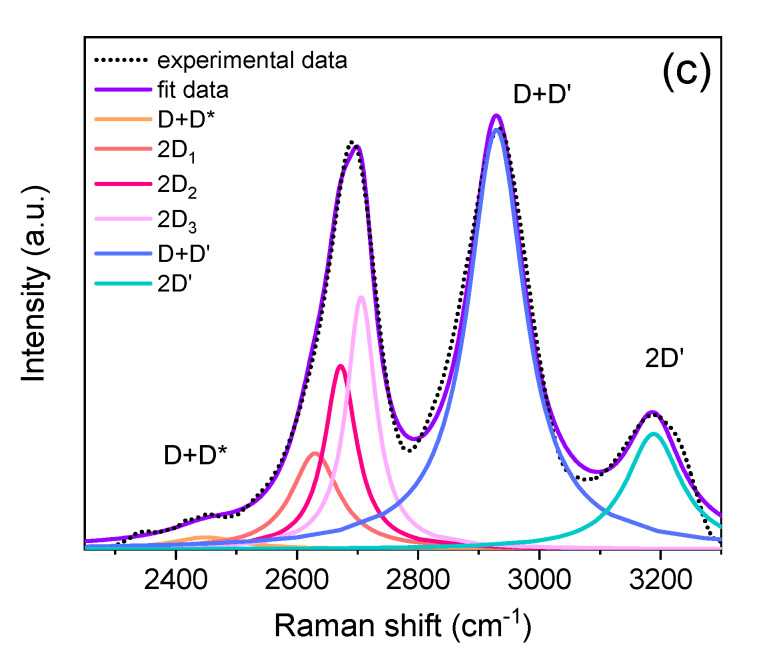
(**a**) Raman spectrum acquired on the as-synthesized graphene nanosheets; (**b**) Deconvolution of the first-order Raman spectrum using five functions (D‘’, D, D_3_, G and D’ bands); (**c**) Deconvolution of the second-order Raman spectrum using six functions (D + D*, 2D_1_, 2D_2_, 2D_3_, D + D’ and 2D’ bands).

The Raman spectrum acquired on the as-exfoliated nanosheets is presented in Figure 2a. It is very similar to the described ones in different works for graphene nanosheets, few-layer graphene or reduced graphene oxide (rGO) [10,25,27]. The typical bands described in literature for these nanomaterials are clearly observed at ~1350 (D), ~1585 (G) and ~2700 (2D) cm^−1^. The G and 2D bands are always found in pristine graphene. The G band is due to the doubly degenerate zone center E2g mode and the 2D band is the second order of zone-boundary phonons [28,29,30]. On the other hand, the D band is the most prominent of the defect-induced bands. It has been reported that these bands arise from breathing-like modes of the carbon rings activated by defects via double-resonance Raman process [28,29,30]. Usually, the I_D_/I_G_ intensity ratio is an indicative of the presence of defects on the graphene lattice [18,31,32].

Along with the peaks that are clearly observed in the spectrum, other defect-induced bands are present at ~1100–1200 and ~1610–1620 cm^−1^. These bands are detected in Figure 2a as a peak that overlaps with the left side of the D band and as a shoulder on the right side of the G peak, respectively. While the latter has been named in most of the published works as D’, the former has been named as T_1_ [33], D_4_ [34], D* [23,27,35] or D’’ [29,30,36] depending on the authors and on the studied carbon-based material. Moreover, a broad shoulder between the D and G peaks can also be seen in Figure 2a. This feature has been related to a Raman band at ~1500 cm^−1^ in defected carbon-based materials, and has been named as T_2_ [33], D_3_ [34,37] or D’’ [27,35] by different authors. This band has been related to the presence of amorphous carbon in graphene oxide [27,35], carbon nanotubes [33] or other carbon-based materials [34]. In the present work, we will assume the nomenclature D‘’, D_3_ and D’ for the bands located at ~1100–1200, ~1500 and ~1610–1620 cm^−1^, respectively.

Usually, the D‘’, D_3_ and D’ bands are not described when analyzing the Raman spectra of graphene-based nanomaterials because they are very weak peaks. Nevertheless, when these bands present a remarkable intensity, they appear to overlap with the D and G peaks. This makes the deconvolution of the first-order spectrum (from 1000 to 2000 cm^−1^) essential for the correct interpretation of the Raman spectrum, as it is suggested by different authors [27,33,35,38,39]. The fittings of the first- and second-order spectra allow the suitable obtaining of the position, intensity (integrated area) and band width of the different peaks. These parameters allow us to establish the presence and nature of defects in the electrochemically exfoliated nanosheets.

Figure 2b,c show examples of the fittings that have been carried out for all the Raman spectra acquired on the electrochemically exfoliated graphene nanosheets, using two Gaussian (D‘’ and D_3_) and three pseudo-Voigt (D, G and D’) functions for the first-order spectra, and three Lorentz (2D) and three pseudo-Voigt (D + D*, D + D’ and 2D´) functions for the second-order spectra.

The high values of the I_D_/I_G_ and I_D’_/I_G_ ratios (Table 1) point to the existence of defects and disorder in the exfoliated nanosheets. Moreover, the low value of I_2D_/I_G_ supports this conclusion, as it has been published that the 2D band of highly disordered graphene reduces its intensity and increases its width [27,31]. Nevertheless, according to the terminology introduced by Ferrari et al. [29] regarding the Raman spectra of disordered graphene, the electrochemically exfoliated nanosheets obtained in the present work would correspond to low-defect graphene (stage I in the classification proposed by these authors). They established a transition between stages I (low-defect graphene) and II (disordered graphene) at I_D_/I_G_ = 3.5, and the intensity ratio of the obtained GNS is lower than this value (2.24 ± 0.05). The existence of a pronounced D_3_ band (see the high value of the I_D3_/I_G_ ratio obtained after fitting, Table 1) is attributed to the presence of amorphous carbon in the nanosheets, as suggested by previous authors [27,34,35].

The shape of the second-order spectrum (Figure 2c)—with a D+D’ band with high intensity—is very similar to the reported one for monolayer graphene bombarded by low-energy argon ions in order to induce disorder in the system [31]. It has been shown that this ion bombardment promotes vacancy-type defects [31,32], so the disorder detected on the exfoliated nanosheets is very likely caused by vacancy-like defects.

Finally, the fitting of the 2D band could be carried out using three Lorentzian functions (Figure 2c), revealing that the GNS present a number of layers lower than 10, according to Ferrari et al. [28] and Malard et al. [40]. Thus, the electrochemical exfoliation technique used in this work allows the production of graphene-like nanosheets: reduced graphene oxide or few-layered defected graphene.

### 3.2. Microstructural Characterization of the Nanocomposite

**Figure 3:**
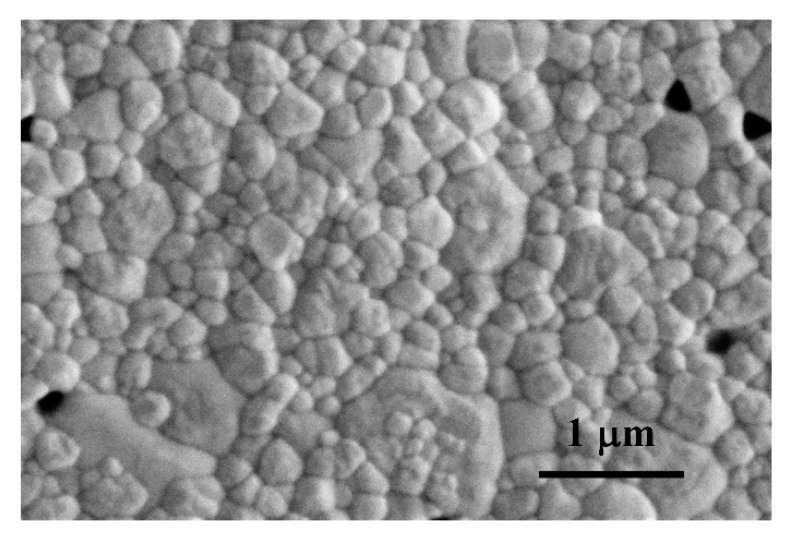
SEM micrograph of the polished c. s. surface of the 3YTZP composite after annealing in air.

A relative density of 99% was obtained for the sintered composite. This high-density value reveals the achievement of a high level of compaction and low porosity in the composite, as it is supported by the SEM micrographs of the composite polished surfaces annealed in air (Figure 3), where pores are not distinguished. It is possible to observe some voids closed to the ceramic grains; however, their size is very similar to that of the grains, which points to the fact that they are the consequence of grain pull-out during the grinding and polishing steps previous to the annealing. The full densification of this type of composites has been previously reported for composites with similar contents of other types of commercial graphene-based nanomaterials, prepared with similar processing and sintering routines [23,41].

A grain size of 0.17 ± 0.09 μm has been obtained for the nanocomposite, revealing a grain refinement with respect to a monolithic 3YTZP ceramic sintered using the same conditions (0.29 ± 0.02 μm [41]), in agreement with the grain growth inhibition effect previously reported for ceramic composites with commercial graphene-based nanomaterials [19,23,25,41]. The grain refinement shown by the composite in this work is more remarkable than the reported ones in previous works for 3YTZP composites with the same content of commercial graphene nanoplatelets [41] (0.27 μm) and graphene nanosheets obtained by mechanical exfoliation of commercial GNP [23] (0.25 μm). This could be related to the optimum GNS distribution throughout the matrix achieved in this work. This is a consequence, on the one hand, of the low dimensions of the electrochemically exfoliated GNS, and, on the other hand, of the adequate use of advanced powder processing and sintering techniques.

**Figure 4:**
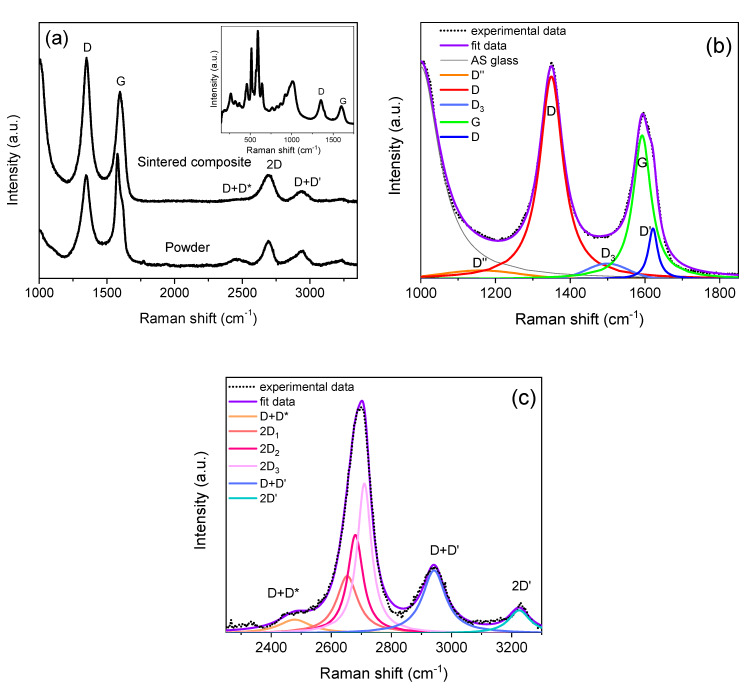
(**a**) Raman spectra acquired on the composite powders after high-energy planetary ball milling and on the sintered composite, inset: detail of the Raman spectrum acquired on the sintered composite in the range 150–1800 cm^−1^; (**b**) Deconvolution of the first-order Raman spectrum of the sintered composite; (**c**) Deconvolution of the second-order Raman spectrum of the sintered composite (D + D*, 2D_1_, 2D_2_, 2D_3_, D + D’ and 2D’ bands).

The Raman spectra of the composite powder after planetary ball milling and of the sintered ceramic composite are presented in Figure 4a. The characteristic peaks for graphene are clearly observed, revealing that neither the high-energy milling during powder processing nor the high temperature during sintering degraded the electrochemically exfoliated GNS. However, a peak with high intensity was detected at ~1000 cm^−1^, which had not been observed in the spectrum of the as-exfoliated GNS (Figure 2a). In order to analyze the origin of this peak, the Raman spectra were acquired in an extended frequency range (inset in Figure 4a) revealing the existence of multiple peaks. Together with the peaks corresponding to the tetragonal (264, 320, 460, 643 cm^−1^) and monoclinic (365, 488 cm^−1^) phases of the zirconia matrix [42], sharp peaks in the range ~500–630 cm^−1^ and a broad band in the range ~700–1100 cm^−1^ were found. These bands can be attributed to the presence of a low percentage of an alumino-silicate (AS) glass [43] that could have been introduced as contamination into the composite powder during the high-energy ball milling. The percentage of this phase must be significantly low, as it was not detected by X-ray diffraction (results not shown). However, future efforts will be carried out to modify the planetary ball milling conditions in order to avoid the formation of this trace of AS glass. In order to perform the deconvolution of the first-order spectra to suitably analyze the defect-related peaks and the intensity ratios, we introduced a new peak—at ~1000 cm^−1^—to the fittings.

Figure 4b,c shows examples of the fittings that have been carried out for all the Raman spectra acquired on the sintered ceramic composite using two Gaussian (D’’ and D_3_) and four pseudo-Voigt (AS glass, D, G and D’) functions for the first-order spectra, and three Lorentz (2D) and three pseudo-Voigt (D + D*, D + D’ and 2D´) functions for the second-order spectra. 

A decrease of the defect-related D and D’ peaks intensity, along with an increase of the intensity of the 2D band, is observed for the GNS in the sintered composite, in comparison with the as-exfoliated GNS (Table 1). Also, a decrease of the D_3_ band is found, pointing to a lower amount of amorphous carbon in the GNS after sintering, in agreement with published results that the I_D3_/I_G_ ratio decreases as the crystallinity increases [27]. All of this reveals a decrease of the number of defects and a restoration of the graphene network during the high-temperature sintering process [27,31,32,39].

Another parameter that can give information about defects in graphene is the band width for D, G, D’ and 2D bands, as their widths increase with a growing number of defects [31]. Martins Ferreira et al. [31] have reported that the width of G and D’ peaks have a less pronounced dependence than the D and 2D bands. According to this assessment, the band widths of the G and D’ peaks stay invariable in both the as-exfoliated GNS and the sintered composite (Table 2), while a lower D band width is clearly observed in the GNS after sintering, which supports the decrease of the number of defects mentioned above. Unexpectedly, the 2D band width stays invariable. However, this parameter is not only dependent on the number of defects, but also on other factors such as doping or strain [3]. It has been published that the 2D band width of graphene subjected to strain suffers a broadening and a shift in frequency [3,44,45]. Table 2 shows the positions of the D, G, D’ and 2D bands, revealing a shift towards higher frequencies for all of them in the spectra of the GNS sintered composite, in comparison to the spectra of the as-exfoliated GNS. This can be attributed to residual stresses in the GNS imposed by the constraining ceramic matrix [44,45]. Thus, the effect of broadening the 2D band as a consequence of the stresses would counteract the decrease of the band width related to the decrease of defects in the GNs after sintering.

**Figure 5:**
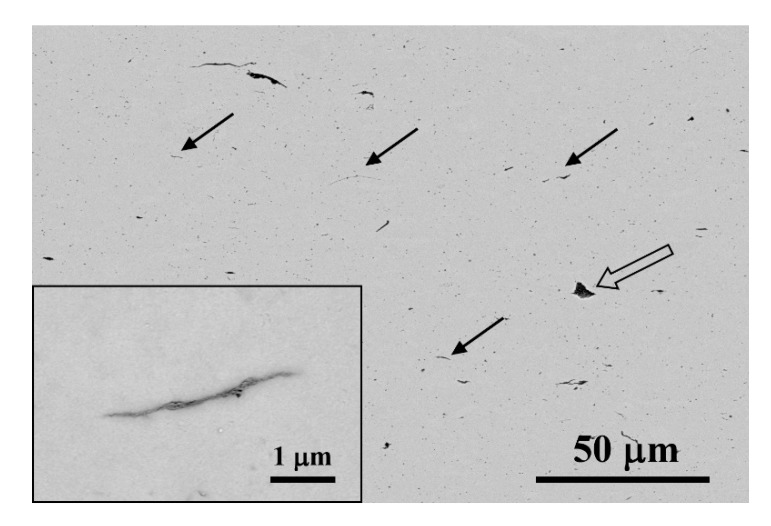
BSE-SEM micrographs of the polished c.s. surface of the sintered composite.

Figure 5 shows the low magnification SEM micrographs acquired on the polished c.s. surface of the nanocomposite using BSE. These images reflect the GNS distribution in the ceramic matrix, as the 3YTZP matrix and the GNS appear in the micrographs as light and dark phases, respectively. A homogeneous distribution of the GNS (marked with thin arrows in the figure) throughout the ceramic matrix is observed, with scarce large GNS agglomerates (marked with a thick arrow). In this c.s. image, most of the observed nanosheets present their side view, which indicates that the ab plane of the graphene layers lies on a plane perpendicular to the compression axis during sintering. This preferential alignment has been previously described for different ceramic composites [19,24,25,41], including composites prepared from powders homogenized using planetary ball milling in wet conditions [20,46]. This structural anisotropy is a consequence of the two-dimensional character of graphene, and the uniaxial pressure applied during the sintering process. When increasing the magnification (inset in Figure 5), very thin GNS with lateral sizes of several microns can be observed throughout the matrix. This could correspond to interconnections of smaller GNS, as previously shown in the HRSEM images of the as-exfoliated GNS (Figure 1b).

**Figure 6:**
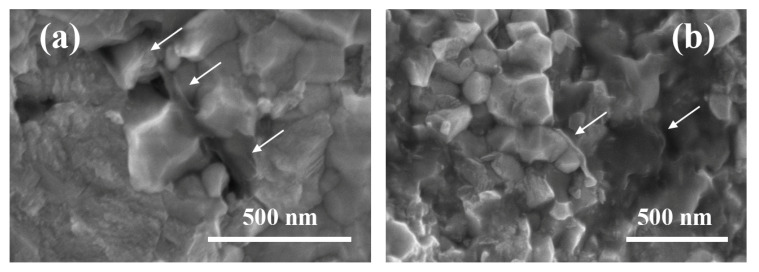
HRSEM micrographs of the fracture surface of the sintered composite. (**a**) Some GNS can be seen as semi-transparent tissue; (**b**) some GNS can be seen from a side view.

The HRSEM images of the fracture surface of the nanocomposite, shown in Figure 6, give an insight into the morphology of the graphene nanosheets incorporated in the 3YTZP matrix. The GNS (marked with arrows) appear as wavy, semi-transparent tissue covering the ceramic grains, as previously reported in ceramic composites with few-layer graphene [25,47]. Some GNS can be seen from a side view, revealing a very low thickness, in accordance with the HRSEM observations of the as-synthesized nanosheets (Figure 1). The fracture surface presents a mostly intergranular fracture mode, which indicates a strong physical bonding at the interphase between the 3YTZP matrix and the GNS, although some areas with intragranular fracture can also be observed.

### 3.3. Vickers Hardness of the Nanocomposite

The Vickers hardness of the nanocomposite, evaluated on i.p. and c.s. surfaces, revealed no mechanical anisotropy, as similar hardness values were obtained for both surfaces (Table 3). This may be due to the small lateral size of the GNS. Also, the values were identical to the reported ones for a monolithic 3YTZP ceramic prepared with similar sintering conditions [41], and very similar to the reported ones for a composite with 1 vol % of graphene nanosheets obtained by exfoliation of commercial graphene nanoplatelets by means of high-energy ball milling [23]. These results indicate that ceramic-based composites containing the electrochemically exfoliated GNS may also display good mechanical strength and fracture toughness. Research work to determine this is already underway.

## 4. Conclusions

Graphene-like nanosheets (GNS) with a number of graphene layers lower than 10, containing amorphous carbon and vacancy-like defects, and with a lateral size lower than 1 μm were successfully synthesized using a simple, cost-effective and fast electrochemical exfoliation method. The incorporation of 1 vol % GNS to a 3YTZP matrix resulted in a composite material with a homogeneous distribution of GNS into the matrix, despite the high tendency to agglomerate presented by the as-exfoliated GNS. The selected processing method—planetary ball milling of the composite powders and spark plasma sintering (SPS)—produced a fully dense nanocomposite containing GNS with significantly enhanced quality, as revealed by Raman spectroscopy. The GNS were wavy, semi-transparent and with very thin thickness. The microstructural anisotropy—preferential alignment of the GNS in the direction perpendicular to the pressing axis during SPS—revealed by SEM observations was not reflected on the hardness values of the nanocomposite, which were isotropic. The hardness of the composite studied here was very similar to the reported one for a 3YTZP composite with the same content of commercial graphene nanosheets.

## Figures and Tables

**Table 1:** Intensity ratios of the D, D_3_, D’ and 2D bands with respect to the G peak, obtained for the as-synthesized graphene-like nanosheets (GNS) and the GNS in the sintered composite after fitting the first- and second-order Raman spectra.

Sample	I_D_/I_G_	I_D3_/I_G_	I_D’_/I_G_	I_2D_/I_G_
As-synthesized GNS	2.24 ± 0.05	0.485 ± 0.021	0.23 ± 0.07	0.219 ± 0.011
Sintered 1 vol % GNS/3YTZP	1.93 ± 0.06	0.249 ± 0.021	0.18 ± 0.07	0.46 ± 0.05

**Table 2:** Positions and band widths of the D, G, D’ and 2D bands obtained for the as-synthesized GNS and the GNS in the sintered composite after fitting the first- and second-order Raman spectra.

Sample	D		G		D’		2D	
	Position (cm^−1^)	Band Width (cm^−1^)	Position (cm^−1^)	Band Width (cm^−1^)	Position (cm^−1^)	Band Width (cm^−1^)	Position * (cm^−1^)	Band Width * (cm^−1^)
As-synthesized GNS	1346.9 ± 0.3	91.5 ± 2.1	1585.34 ± 1.02	52 ± 1	1617.5 ± 0.7	27.2 ± 0.9	2687.6 ± 0.4	103.96 ± 1.6
Sintered 1 vol % GNS/3YTZP	1350.3 ± 0.4	75.3 ± 2.4	1592.1 ± 1.3	53 ± 2	1621.8 ± 0.5	32.7 ± 1.3	2690.9 ± 1.1	105 ± 3

* Values obtained after fitting the 2D band to a pseudo-Voigt function (not shown).

**Table 3:** Vickers hardness for the composite in this study, compared with a monolithic 3YTZP ceramic and a composite prepared with commercial graphene nanosheets.

Sample	H_i.p._ (GPa)	H_c.s._ (GPa)
3YTZP [41]	13.9 ± 0.5	
1 vol % GNS/3YTZP (this work)	14.00 ± 0.13	13.9 ± 0.8
1 vol % GNS/3YTZP [23]	13.6 ± 0.8	12.7 ± 0.7
